# Characterization of the HIV-1 RNA associated proteome identifies Matrin3 and PSF as nuclear cofactors of Rev function

**DOI:** 10.1186/1742-4690-8-S2-P42

**Published:** 2011-10-03

**Authors:** Anna Kula, Jessica Guerra, Maryana Bardína, Alessandro Marcello

**Affiliations:** 1 Laboratory of Molecular Virology, International Centre for Genetic Engineering and Biotechnology (ICGEB), Padriciano, 99, 34149 Trieste, Italy; 2 Department of Microbiology and Molecular Medicine, University of Geneva, Geneva, 1211, Switzerland

## Background

Generation of infectious HIV-1 requires the synthesis of the spliced and the Rev responsive element (RRE)-containing viral transcripts. The former is efficiently processed and rapidly exported to the cytoplasm. The latter retains introns in unspliced and partially spliced forms and does not exit the nucleus until the viral Rev protein mediates its export for the expression of structural proteins and for the production of full-length genomic RNAs. Rev by binding to RRE increases the half- life of unspliced/ partially spliced RNAs and promotes their export to the cytoplasm. Various cellular factors have been reported to contribute to Rev activities but the molecular details of how Rev overcomes the eukaryotic restriction for intron-containing mRNAs remain to be determined.

## Materials and methods

Here we introduced a novel method to explore the proteome associated with the nuclear HIV-1 RNAs. At the core of the method is the generation of cell lines harboring an integrated HIV-1 provirus carrying RNA binding sites for the MS2 bacteriophage protein. Flag-tagged MS2 was then used for affinity purification of the viral RNA.

## Results

In the proteomic screen we found that the viral RNA is associated with the host nuclear matrix component MATR3 and the polypyrimidine tract-binding protein associated splicing factor (PSF) 1. MATR3 has been recently identified as a cofactor of Rev function [1,2] and PSF was shown previously to associate with HIV-1 RNA [3]. We showed that knockdown of either factor inhibited Rev activity. While MATR3 depletion suppressed the Rev-dependent export of HIV-1 RNAs, PSF depletion affected the nuclear stability of Rev-dependent transcripts. Further we showed that MATR3 and PSF interact with each other and are able to associate with Rev.

## Conclusions

We provide evidence that MATR3 and PSF are novel nuclear cofactors that control Rev activity. We speculate that they are components of a nuclear pathway hijacked by Rev to facilitate the nuclear survival and export of intron-containing viral transcripts.
